# Modulatory Effects of Procyanidin B1 on Inflammation-Induced Oxidative Stress and ECM-Related Responses in Human Dermal Fibroblasts and Epidermal Keratinocytes

**DOI:** 10.3390/molecules31132294

**Published:** 2026-07-01

**Authors:** Sullim Lee, Baolin Zhu, Daeyoung Kim, Dae Sik Jang

**Affiliations:** 1 Department of Life Science, College of Bio-Nano Technology, Gachon University, Seongnam 13120, Republic of Korea; sullimlee@gachon.ac.kr; 2 Department of Fundamental Pharmaceutical Sciences, Graduate School, Kyung Hee University, Seoul 02447, Republic of Korea; zhubaolin416@khu.ac.kr; 3 College of Pharmacy, Kyung Hee University, 26 Kyungheedae-ro, Dongdaemun-gu, Seoul 02447, Republic of Korea

**Keywords:** procyanidin B1, *Nypa fruticans*, skin aging, oxidative stress, ECM degradation

## Abstract

Oxidative stress and inflammation are central environmental contributors to skin aging, accelerating extracellular matrix (ECM) breakdown and loss of dermal structure. Although *Nypa fruticans* is recognized for its antioxidant properties, the constituents responsible for these effects remain undefined. To address this, we screened five major polyphenols—protocatechuic acid (PA), hydroxybenzoic acid (HA), procyanidin B1 (PB), catechin (CA), and epicatechin (EC)—for protective activity in two inflammatory skin cell models: human dermal fibroblasts (HDFs) stimulated with tumor necrosis factor-α (TNF-α), and human epidermal keratinocytes (HEKs) co-stimulated with TNF-α and interferon-γ (IFN-γ). PB emerged as the most consistently active compound. In fibroblasts, it suppressed intracellular reactive oxygen species, limited matrix metalloproteinase-1 (MMP-1) release, and restored pro-collagen I α1 output. In keratinocytes, it reduced both pro-inflammatory cytokines—interleukin (IL)-6, IL-8, and IL-1β and inflammatory mediators, including prostaglandin E_2_ (PGE_2_), cyclooxygenase-2 (COX-2), and nitric oxide (NO). At the transcriptional level, PB shifted the ECM balance by lowering MMP expression while elevating collagen- and hyaluronan-associated genes. Collectively, these results position PB as a principal driver of the protective activity of *Nypa fruticans* (*N. fruticans*) leaves under inflammatory conditions. Mechanistically, PB suppressed nuclear factor kappa B (NF-κB) activation and promoted nuclear factor erythroid 2-related factor 2 (Nrf2) nuclear translocation in keratinocytes, supporting its dual anti-inflammatory and antioxidant activities.

## 1. Introduction

Intracellular reactive oxygen species (ROS) are central to the development of numerous human diseases, where they inflict oxidative damage on cellular and molecular components [1,2]. Such reactive species arise chiefly during mitochondrial oxidative phosphorylation, as electrons escaping the transport chain react with molecular oxygen [3]. Under healthy conditions, redox balance is sustained by endogenous antioxidant defenses, including superoxide dismutase (SOD), catalase, and glutathione peroxidase, which scavenge surplus ROS before damage occurs [4]. However, once ROS generation outpaces this protective capacity, oxidative stress ensues and drives the onset of diverse pathologies, including neurodegenerative disorders, cardiovascular disease, and skin aging [5].

The skin is among the organs most vulnerable to oxidative injury owing to its constant contact with environmental stressors, such as ultraviolet (UV) radiation and airborne pollutants [6]. Cutaneous aging is generally divided into intrinsic aging, reflecting time-dependent cellular decline, and extrinsic aging, driven largely by external factors, with UV radiation being the foremost [7]. Excessive accumulation of ROS accelerates aging by oxidatively damaging core extracellular matrix (ECM) constituents, promoting fragmentation and aberrant cross-linking of fibrous proteins such as collagen, elastic fibers, and glycosaminoglycans [8,9]. This deterioration is compounded by the ROS-driven activation of collagen-degrading enzymes, most notably matrix metalloproteinase-1 (MMP-1) [10]. Pro-inflammatory cytokines, including tumor necrosis factor-α (TNF-α) and interferon-γ (IFN-γ), induce ECM degradation by upregulating matrix metalloproteinases (particularly MMP-1) while suppressing collagen synthesis, thereby shifting tissue remodeling toward net matrix loss and accelerating skin aging [11].

To mitigate these effects, various natural antioxidants have been investigated for their potential to alleviate photoaging and oxidative stress–induced skin damage. Polysaccharides isolated from *Spirulina platensis*, for example, were found to lower ROS levels and suppress MMP expression [12]. For example, bioactive peptides derived from skipjack tuna (*Katsuwonus pelamis*), including TCP3 (PKK), TCP6 (YEGGD), and TCP9 (GPGLM), have been reported to alleviate UV-induced skin damage by reducing oxidative stress and protecting skin cells [13]. Likewise, extracts of *Chrysanthemum morifolium* have been documented to ease ultraviolet B (UVB)-induced skin damage via antioxidant and anti-inflammatory actions [14]. Similarly, an ethanol extract of *Hibiscus sabdariffa* exhibited notable antioxidant and anti-aging activities in cell-based assays, supporting the dermatological potential of polyphenol-rich botanicals [15]. Despite these advances, most studies have focused on individual compounds without a comparative evaluation of their relative contributions to the overall effect. Moreover, studies investigating underutilized botanical sources with high polyphenol content and potential dermatological benefits remain limited.

Traditionally, *Nypa fruticans* has been used to manage asthma, rheumatism, and pain, and its pharmacological properties have recently drawn increasing scientific attention [16]. Notably, it has demonstrated antioxidant and anti-diabetic activities, suggesting potential therapeutic applications in oxidative stress-related conditions and metabolic disorders [17]. Previous studies have highlighted its ability to reduce oxidative stress and metabolic dysfunction [18]. Our earlier work showed that in keratinocytes, *N. fruticans* extract lowered intracellular ROS and MMP-1 expression while raising hyaluronan synthase levels (*HAS-1* and *HAS-2*), thereby blunting TNF-α-driven oxidative stress and ECM-related injury [19]. Taken together, this study positioned the extract as a contributor to ECM integrity and skin hydration.

Chemical analysis identified five major polyphenolic constituents in *N. fruticans*: protocatechuic acid (PA), hydroxybenzoic acid (HA), procyanidin B1 (PB), catechin (CA), and epicatechin (EC). These compounds were selected for comparative evaluation because they represent the predominant polyphenolic components of the extract and may differentially contribute to its biological activity. Although the antioxidant and anti-inflammatory activities of some of these compounds, including PB, have been previously reported, the relative contributions and comparative efficacies of the major polyphenols derived from *N. fruticans* under inflammatory conditions relevant to skin aging remain unclear. In particular, direct comparative studies systematically evaluating the relative bioactivities of these compounds in skin cell models are limited.

Therefore, the present study aimed to address this gap using a direct comparative approach by systematically evaluating the antioxidant, anti-inflammatory, and ECM-regulatory effects of the five major polyphenols in inflammatory skin cell models. Using TNF-α-stimulated human dermal fibroblasts (HDFs) and TNF-α/IFN-γ-stimulated human epidermal keratinocytes (HEKs), we sought to identify the compound showing the most consistent protective activity, determine the relative contribution of each major constituent to the biological effects of *N. fruticans,* and investigate their effects on oxidative stress, inflammation, and ECM-related responses.

## 2. Results

### 2.1. Cell Viability of PA, HA, PB, CA, and EC Derived from N. fruticans

**Figure 1:**
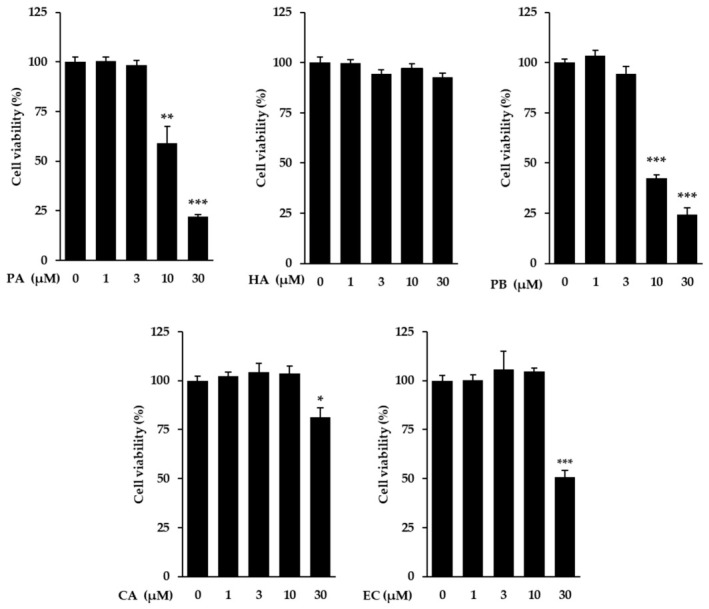
Cytotoxic effects of PA, HA, PB, CA, and EC on HDFs. Cells were plated in 96-well plates at a density of 1 × 10^4^ cells/well and grown for 24 h. The medium was then switched to serum-free DMEM to impose nutrient deprivation, and the cells were maintained for a further 24 h. Increasing concentrations of each compound were applied for 24 h, after which cell viability was assessed using the EZ-Cytox assay. Values represent the mean ± SEM of three independent experiments, with significance determined against the vehicle control (* *p* < 0.05, ** *p* < 0.01, *** *p* < 0.001). PA, protocatechuic acid; HA, hydroxybenzoic acid; PB, procyanidin B1; CA, catechin; EC, epicatechin; HDFs, human dermal fibroblasts; DMEM, Dulbecco’s modified Eagle’s medium; SEM, standard error of the mean.

A preliminary cell viability assay was performed on HDFs to evaluate the cytotoxicity of five major polyphenols derived from *N. fruticans*: PA, HA, PB, CA, and EC. As depicted in Figure 1, exposure to 10 μM PA and PB sharply lowered cell viability to 59.2% and 42.3%, respectively. At a higher concentration (30 μM), cell viability was further reduced by PA (22.1%), PB (24.5%), CA (81.2%), and EC (50.6%), indicating concentration-dependent cytotoxic effects. In contrast, HA exhibited minimal cytotoxicity under the tested conditions. Based on these results, concentrations ≤ 3 μM were selected for all subsequent functional assays to minimize potential cytotoxic effects while allowing the detection of compound-specific biological activity.

### 2.2. Effects of PA, HA, PB, CA, and EC on Intracellular and Mitochondrial ROS Generation in TNF-α-Stimulated HDFs

**Figure 2:**
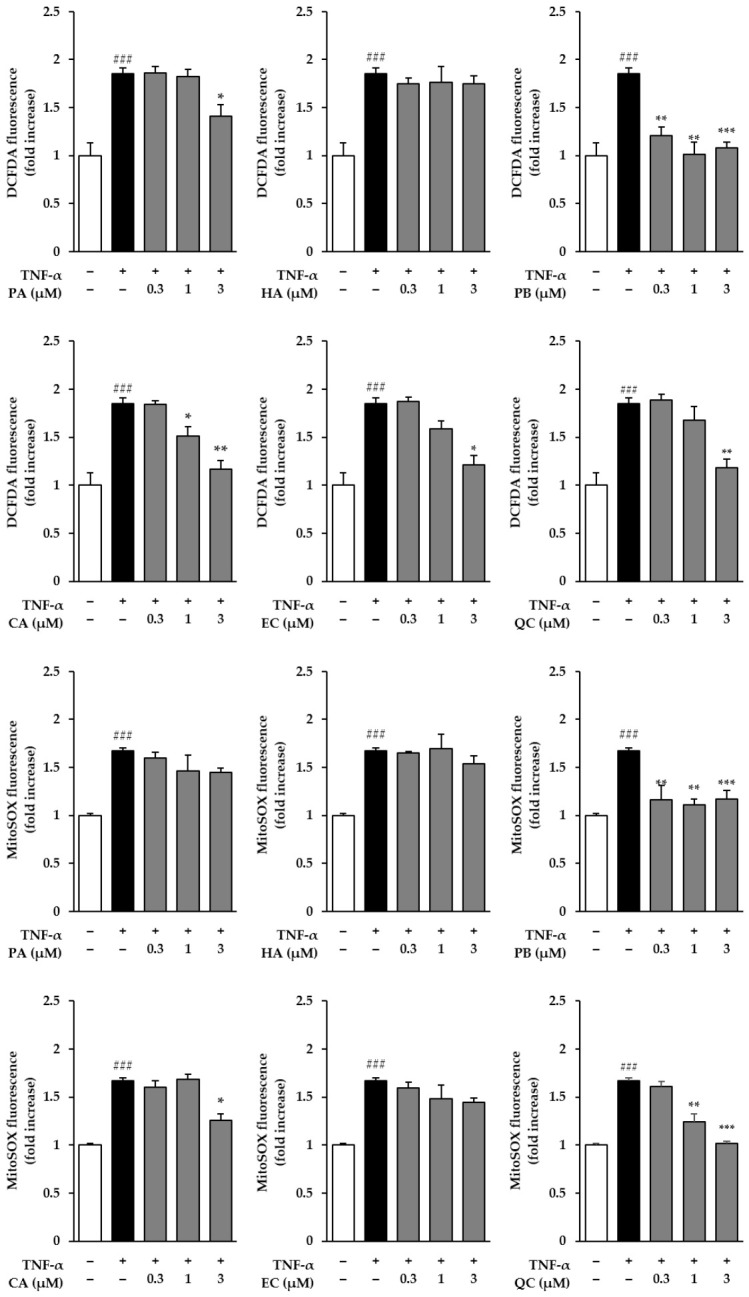
Effects of PA, HA, PB, CA, and EC on intracellular and mitochondrial ROS production in HDFs. Cells were dispensed into black 96-well plates at 1 × 10^4^ cells/well and grown for 24 h, after which the medium was replaced with serum-free DMEM for a further 24 h. The cells were treated with the indicated compounds for 1 h and then exposed to TNF-α (20 ng/mL) for 15 min. Intracellular ROS was recorded by DCFDA staining (upper panels) and mitochondrial ROS by MitoSOX staining (lower panels), with fluorescence read on a microplate reader. QC, a well-characterized antioxidant known to limit ROS accumulation, was used as a positive control. Values are expressed as the mean ± SEM of three independent experiments. ^###^ *p* < 0.001 vs. control; * *p* < 0.05, ** *p* < 0.01, *** *p* < 0.001 vs. the TNF-α-only group. ROS, reactive oxygen species; TNF-α, tumor necrosis factor-α; DCFDA, 2′,7′-dichlorofluorescein diacetate; QC, quercetin.

To evaluate the antioxidant potential of the tested compounds, intracellular and mitochondrial ROS levels were measured in HDFs following stimulation with TNF-α using DCFDA and MitoSOX assays, respectively. Quercetin (QC), a natural antioxidant that suppresses ROS accumulation in fibroblasts, was used as a positive control [20]. As shown in Figure 2, TNF-α treatment markedly increased both intracellular and mitochondrial ROS production compared to the untreated control group (^###^ *p* < 0.001), indicating the induction of oxidative stress under inflammatory conditions.

Pre-treatment with PA, PB, CA, and EC attenuated TNF-α-induced intracellular ROS accumulation to varying degrees. Among these compounds, PB exhibited the most consistent antioxidant effect, whereas EC exhibited strong inhibitory activity in the DCFDA assay. PA and CA exhibited moderate ROS-suppressive effects, whereas HA did not significantly reduce intracellular ROS generation.

Additional evaluation using MitoSOX revealed that PB markedly reduced mitochondrial ROS generation in TNF-α-stimulated HDFs, suggesting that its antioxidant activity extends to the regulation of mitochondrial oxidative stress. As a positive control, QC also significantly attenuated mitochondrial ROS production. PA and HA had limited effects, whereas CA moderately suppressed at the highest concentration tested. In contrast, EC showed weaker effects in the MitoSOX assay than in the DCFDA assay, suggesting that its antioxidant activity may involve mechanisms beyond the direct suppression of mitochondrial ROS.

These findings indicate that *N. fruticans*-derived polyphenols differentially regulate oxidative stress depending on the origin of ROS and the cellular compartment. Among the tested compounds, PB demonstrated the strongest and most consistent suppressive effects on both intracellular and mitochondrial ROS generation, supporting its superior antioxidant potential in inflammatory conditions.

### 2.3. Effects of PA, HA, PB, CA, and EC on MMP-1 Secretion in TNF-α-Stimulated HDFs

**Figure 3:**
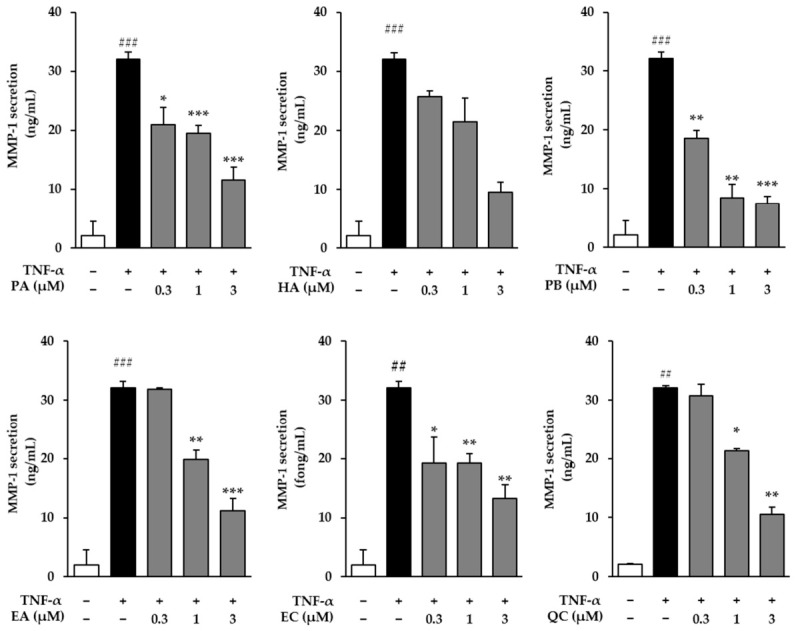
Effects of PA, HA, PB, CA, and EC on TNF-α-induced MMP-1 secretion in HDFs. Cells were distributed into 48-well plates at 2 × 10^4^ cells/well and held for 24 h; the medium was then changed to serum-free DMEM, and the cells were kept for another 24 h before treatment. After 1 h of exposure to each compound, the cells were challenged with TNF-α (20 ng/mL) for 24 h. Conditioned media were harvested, and MMP-1 was measured using ELISA, with results given as absolute MMP-1 concentrations (ng/mL). QC was used as a reference antioxidant. Data are presented as means ± SEM of two independent experiments. ^##^ *p* < 0.01, ^###^ *p* < 0.001 vs. vehicle control; * *p* < 0.05, ** *p* < 0.01, *** *p* < 0.001 vs. the TNF-α-treated group. MMP-1, matrix metalloproteinase-1; ELISA, enzyme-linked immunosorbent assay.

To determine whether the tested compounds modulate ECM-related responses under inflammatory conditions, the effects of each compound on TNF-α-induced MMP-1 secretion were evaluated in HDFs using an ELISA. As shown in Figure 3, stimulation with TNF-α markedly increased MMP-1 secretion compared that to in the unstimulated control group. The basal MMP-1 level in untreated HDFs was 2.05 ± 2.50 ng/mL, whereas TNF-α treatment increased MMP-1 secretion to 32.08 ± 1.11 ng/mL, corresponding to an approximately 15.6-fold increase compared to the control group (^###^ *p* < 0.001). These results indicate that TNF-α effectively induces an ECM-degrading response in HDFs.

Pre-treatment with the tested polyphenols reduced TNF-α-induced MMP-1 secretion to varying extents. PA treatment resulted in a concentration-dependent reduction, reaching 11.60 ± 2.21 ng/mL at 3 μM, corresponding to a 63.8% decrease relative to the TNF-α-only group (*** *p* < 0.001). HA showed comparatively weaker activity at lower concentrations but reduced MMP-1 secretion to 9.53 ± 1.74 ng/mL at 3 μM (70.3% reduction).

Among the tested compounds, PB exhibited the most potent inhibitory effect on MMP-1 secretion. Treatment with PB decreased MMP-1 levels from 32.08 ± 1.11 ng/mL to 18.63 ± 1.31 ng/mL, 8.43 ± 2.40 ng/mL, and 7.48 ± 1.19 ng/mL at 0.3, 1, and 3 μM, respectively, corresponding to reductions of 41.9%, 73.7%, and 76.7% compared with TNF-α treatment alone (* *p* < 0.01–0.001). PB treatment at 1 and 3 μM reduced MMP-1 secretion to levels approaching those observed under non-inflammatory conditions.

CA and EC also suppressed MMP-1 secretion, but their response profiles differed from that of PB. CA reduced MMP-1 secretion to 11.15 ± 2.13 ng/mL at 3 μM (65.3% reduction), whereas EC reduced secretion to 13.26 ± 2.34 ng/mL at the same concentration (58.7% reduction). In both cases, a significant inhibition was observed at higher concentrations.

Overall, all tested compounds, except HA, showed measurable suppression of TNF-α-induced MMP-1 secretion, although the magnitude of inhibition varied among the compounds. PB consistently demonstrated the greatest reduction across all tested concentrations, followed by CA and EC.

### 2.4. Effects of PA, HA, PB, CA, and EC on Pro-Collagen I α1 Secretion in TNF-α-Stimulated HDFs

**Figure 4:**
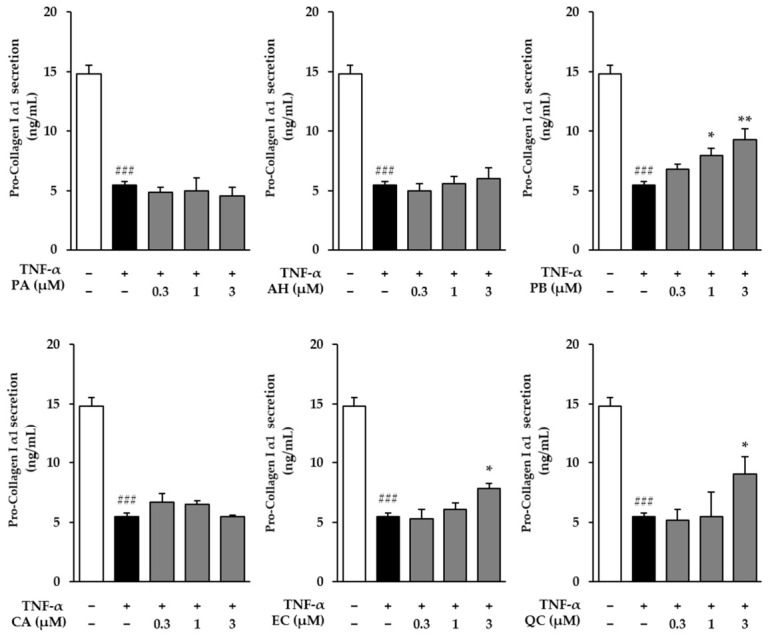
Effects of PA, HA, PB, CA, and EC on pro-collagen I α1 secretion in TNF-α-stimulated HDFs. Cells were placed in 48-well plates at 2 × 10^4^ cells/well and maintained for 24 h, then transferred to serum-free DMEM for another 24 h prior to treatment. Following a 1 h pre-incubation with each compound, the cells were stimulated with TNF-α (20 ng/mL) for 24 h. Pro-collagen I α1 levels in the collected supernatants were quantified using ELISA and reported as absolute concentrations (ng/mL). Quercetin (QC) was used as the reference antioxidant. The results are expressed as the mean ± SEM of two independent experiments. ^###^ *p* < 0.001 versus vehicle control; * *p* < 0.05, ** *p* < 0.01 versus TNF-α-treated group.

To further evaluate the effects of the tested compounds on ECM-related responses, pro-collagen I α1 secretion was measured in TNF-α-stimulated HDFs using ELISA. As shown in Figure 4, TNF-α stimulation significantly reduced pro-collagen I α1 secretion compared that to in the untreated control group. The basal pro-collagen I α1 level in untreated HDFs was 14.79 ± 0.74 ng/mL, whereas TNF-α treatment reduced the secretion to 5.47 ± 0.30 ng/mL, corresponding to a decrease of approximately 63.0% relative to the control group (^###^ *p* < 0.001). These results indicate that inflammatory stimulation markedly impairs collagen-related responses in HDFs.

Among the tested compounds, PB exhibited the most prominent restorative effect on pro-collagen I α1 secretion. Treatment with PB increased pro-collagen I α1 levels from 5.47 ± 0.30 ng/mL to 6.80 ± 0.44 ng/mL, 7.99 ± 0.59 ng/mL, and 9.32 ± 0.89 ng/mL at 0.3, 1, and 3 μM, respectively, corresponding to recovery of 46.0%, 54.0%, and 63.0% of the control levels. Significant restoration was observed at 1 and 3 μM (*^–^** *p* < 0.05–0.01 vs. TNF-α-treated group).

EC showed a weaker but detectable restorative effect, reaching 7.84 ± 0.44 ng/mL at 3 μM, corresponding to approximately 53.0% of the control level (* *p* < 0.05). In contrast, PA, HA, and CA did not significantly restore pro-collagen I α1 secretion under the tested conditions, despite minor changes in the absolute concentration.

Overall, the tested polyphenols exhibited differential effects on collagen-related responses in inflammatory conditions. PB consistently exhibited the greatest ability to restore pro-collagen I α1 secretion following TNF-α stimulation.

### 2.5. Integrated Analysis of PA, HA, PB, CA, and EC Using Radar Chart Visualization in TNF-α-Stimulated HDFs

To comprehensively compare the relative efficacy of the five *N. fruticans*-derived polyphenols, an integrated analysis was performed using radar chart visualization based on three key parameters: inhibition of intracellular ROS generation, suppression of MMP-1 secretion, and restoration of pro-collagen I α1 production. These parameters were selected to reflect oxidative stress, ECM degradation, and ECM synthesis.

**Figure 5:**
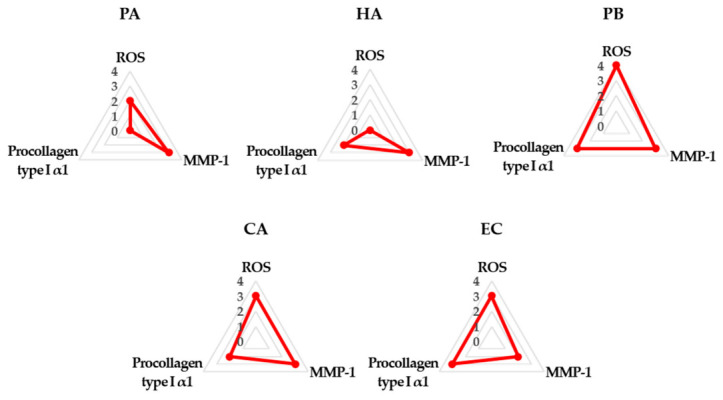
Radar plot summarizing the integrated efficacy of PA, HA, PB, CA, and EC in TNF-α-stimulated HDFs. Each compound was scored for intracellular ROS inhibition, MMP-1 suppression, and pro-collagen I α1 restoration using a semi-quantitative scoring system (0–4). The red polygon represents the integrated activity profile of each compound, and a larger polygon indicates a greater overall biological efficacy. The detailed scoring criteria are described in the Materials and Methods section.

As shown in Figure 5, PB exhibited the highest overall performance across all three parameters, demonstrating consistent activity in reducing ROS levels, suppressing MMP-1 secretion and enhancing pro-collagen I α1 production. EC and CA also showed measurable activity; however, their effects were more limited or parameter-specific than those of PB. In contrast, PA and HA displayed relatively weaker activity across the evaluated conditions, highlighting the non-uniform distribution of the biological activities of the major polyphenols.

This integrated comparison highlights that PB is the polyphenol with the most balanced and consistent effects. Based on these results, PB was selected for subsequent analyses to further investigate its effects on inflammatory responses and ECM-related gene expression levels.

### 2.6. Effects of PB on IL-6, IL-8, and IL-1β Secretion in HEKs Induced by TNF-α/IFN-γ

**Figure 6:**
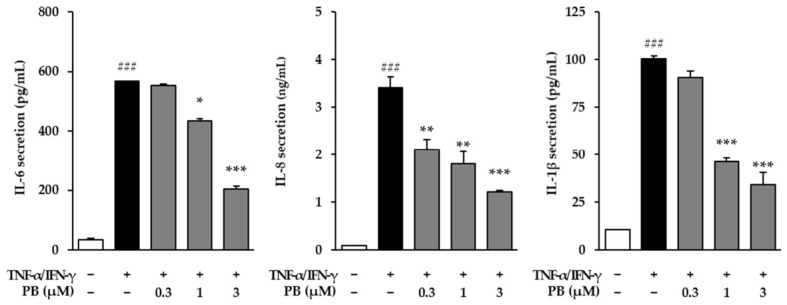
Effects of PB on TNF-α/IFN-γ-induced secretion of pro-inflammatory cytokines in HEKs. Cells were plated in 48-well plates at 2 × 10^4^ cells/well and cultured for 24 h, then shifted to serum-free DMEM under nutrient-poor conditions for 24 h. After a 1 h pretreatment with PB, cells were co-stimulated with TNF-α (20 ng/mL) and IFN-γ (20 ng/mL) for 12 h. IL-6, IL-8, and IL-1β levels in the supernatants were measured using ELISA. Bars indicate the mean ± SEM of two independent experiments. ^###^ *p* < 0.001 vs. untreated control; * *p* < 0.05, ** *p* < 0.01, *** *p* < 0.001 vs. TNF-α/IFN-γ-treated group. HEKs, human epidermal keratinocytes; IFN-γ, interferon-γ; IL-6, interleukin-6; IL-8, interleukin-8; IL-1β, interleukin-1β.

To assess whether PB curbs cytokine release, the secreted amounts of the pro-inflammatory cytokines interleukin-6 (IL-6), interleukin-8 (IL-8), and interleukin-1β (IL-1β) were quantified in HEKs challenged with TNF-α/IFN-γ. Dual cytokine treatment increased the release of all three cytokines well above the control levels (^###^ *p* < 0.001; Figure 6), verifying that an inflammatory response had been established under these conditions.

PB treatment significantly reduced the secretion of IL-6, IL-8, and IL-1β in a concentration-dependent manner. Notably, PB at 3 μM markedly suppressed the levels of all three cytokines compared to the TNF-α/IFN-γ-treated group (*** *p* < 0.001). This inhibitory effect was consistently observed across all tested cytokines, indicating that PB modulates cytokine production under inflammatory conditions.

Taken together with the observed reduction in ROS production and MMP-1 secretion, these findings suggest that PB regulates both oxidative stress and inflammation-related responses in TNF-α/IFN-γ-stimulated HEKs.

### 2.7. Effects of PB on Inflammatory Mediator Production in TNF-α/IFN-γ-Stimulated HEKs

**Figure 7:**
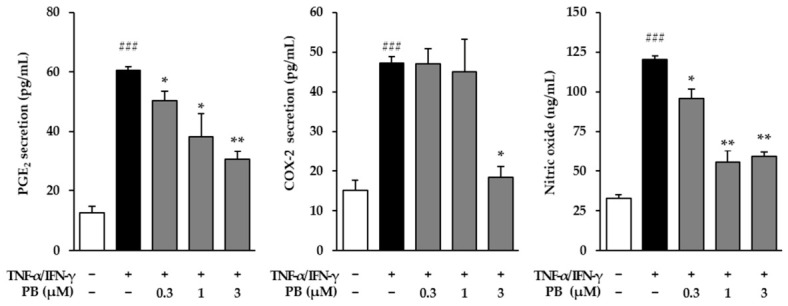
Effects of PB on TNF-α/IFN-γ-induced inflammatory mediators in HEKs. For PGE_2_ and COX-2, cells were seeded in 48-well plates at 2 × 10^4^ cells/well, allowed to settle for 24 h, and then starved in serum-free DMEM for 24 h. After a 1 h PB pre-treatment, cells were co-stimulated with TNF-α (20 ng/mL) and IFN-γ (20 ng/mL) for 24 h, and PGE_2_ and COX-2 levels in the supernatants were measured using ELISA. For NO, cells were arrayed in 96-well plates at 1 × 10^4^ cells/well and handled identically, with NO determined using the Griess method. Data are presented as the mean ± SEM of two (PGE_2_ and COX-2) or three (NO) independent experiments. ^###^ *p* < 0.001 vs. control group; * *p* < 0.05, ** *p* < 0.01 vs. TNF-α/IFN-γ-only group. PGE_2_, prostaglandin E2; COX-2, cyclooxygenase-2; NO, nitric oxide.

To further characterize the anti-inflammatory effects of PB, levels of representative inflammatory mediators—prostaglandin E_2_ (PGE_2_), cyclooxygenase-2 (COX-2), and nitric oxide (NO)—were assessed in keratinocytes stimulated with TNF-α and IFN-γ. As shown in Figure 7, co-stimulation with TNF-α and IFN-γ significantly increased the levels of PGE_2_ and COX-2, along with a marked elevation in NO production compared to the untreated control group (^###^ *p* < 0.001), indicating the activation of inflammatory signaling.

Treatment with PB reduced the production of these inflammatory mediators, with varying degrees of efficacy. PGE_2_ levels were significantly decreased at all tested concentrations, indicating a consistent inhibitory effect of the treatment. COX-2 levels were also reduced, with a statistically significant decrease observed at 3 μM (* *p* < 0.05). In addition, NO production was significantly attenuated in a concentration-dependent manner (** *p* < 0.01), suggesting that PB suppresses NO generation under inflammatory conditions.

These findings indicate that PB modulates multiple inflammatory mediators induced by TNF-α/IFN-γ stimulation. Together with the observed reductions in pro-inflammatory cytokines (Section 2.6), these results support the role of PB in regulating inflammatory responses in HEKs.

### 2.8. Effects of PB on NF-κB and Nrf2 Signaling in TNF-α/IFN-γ-Stimulated Human Epidermal Keratinocytes

**Figure 8:**
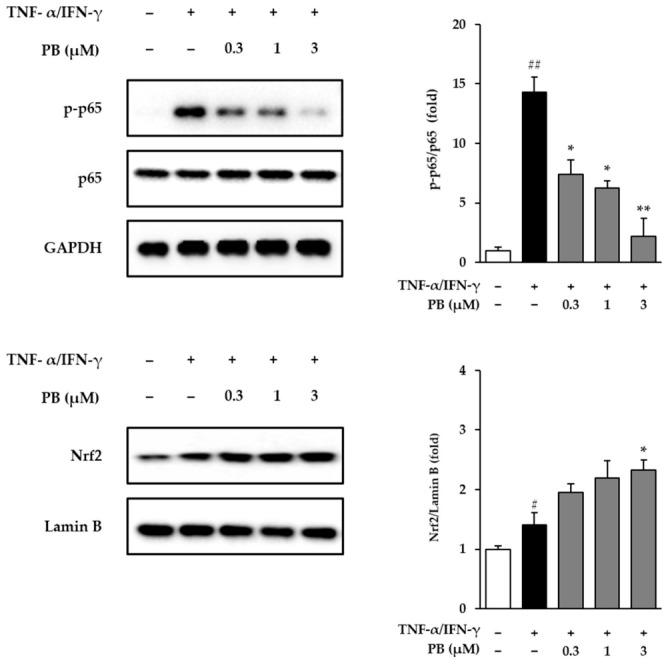
Effects of PB on NF-κB and Nrf2 signaling in TNF-α/IFN-γ-stimulated HEKs. Cells were pretreated with PB (0.3, 1, or 3 μM) for 1 h and then stimulated with TNF-α (20 ng/mL) and IFN-γ (20 ng/mL). Phosphorylated p65 (p-p65) and total p65 were analyzed in whole-cell lysates using western blotting, with GAPDH as a loading control. Nuclear Nrf2 was analyzed in nuclear fractions using Lamin B as a nuclear loading control. Band intensities were quantified by densitometry and expressed as fold of control (p-p65/p65 and Nrf2/Lamin B). Data are presented as the mean ± SEM from two independent experiments. ^#^ *p* < 0.05, ^##^ *p* < 0.01 vs. control; * *p* < 0.05, ** *p* < 0.01 vs. the TNF-α/IFN-γ-treated group. NF-κB, nuclear factor-κB; Nrf2, nuclear factor erythroid 2–related factor 2.

To elucidate the signaling mechanisms underlying the anti-inflammatory and antioxidant effects of PB, the nuclear factor kappa B (NF-κB) and nuclear factor erythroid 2-related factor 2 (Nrf2) pathways were examined using western blotting in TNF-α/IFN-γ-stimulated HEKs. As shown in Figure 8, co-stimulation with TNF-α and IFN-γ markedly increased the phosphorylation of p65 (p-p65), with the p-p65/p65 ratio rising to 14.32 ± 1.25-fold of the control level (^##^ *p* < 0.01), indicating the activation of NF-κB signaling. Pre-treatment with PB suppressed p-p65 in a concentration-dependent manner, decreasing the p-p65/p65 ratio to 7.41 ± 1.18, 6.24 ± 0.64, and 2.19 ± 1.51-fold at 0.3, 1, and 3 μM, respectively (* *p* < 0.05, ** *p* < 0.01 vs. the TNF-α/IFN-γ-treated group), whereas total p65 levels remained unchanged.

Simultaneously, the effect of PB on Nrf2 was evaluated using nuclear fractions. Nuclear Nrf2 levels, normalized to Lamin B, were increased by PB treatment in a concentration-dependent manner, rising from 1.41 ± 0.20-fold (TNF-α/IFN-γ alone) to 1.95 ± 0.15, 2.20 ± 0.29, and 2.33 ± 0.17-fold of the control level at 0.3, 1, and 3 μM, respectively (* *p* < 0.05 vs. the TNF-α/IFN-γ-treated group), indicating enhanced nuclear translocation of Nrf2. These results demonstrate that PB suppresses NF-κB activation while promoting Nrf2-mediated antioxidant signaling, providing a mechanistic basis for the reduction in pro-inflammatory mediators and reactive oxygen species observed in the preceding experiments.

### 2.9. Effects of PB on ECM Gene mRNA Expression in HEKs

**Figure 9:**
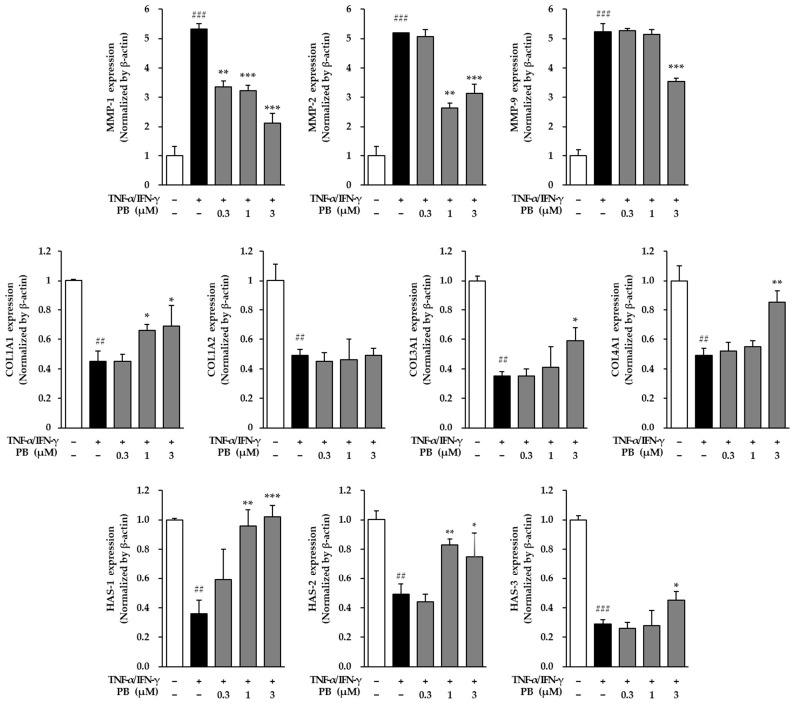
Effects of PB on ECM-related gene expression in TNF-α/IFN-γ-stimulated HEKs. Cells were pre-incubated with PB (0.3, 1, or 3 μM) for 1 h and then co-stimulated with TNF-α (20 ng/mL) and IFN-γ (20 ng/mL) for 12 h. Total RNA was extracted, and transcript levels of matrix metalloproteinases (*MMP-1*, *MMP-2*, *MMP-9*), collagen genes (*COL1A1*, *COL1A2*, *COL3A1*, *COL4A1*), and hyaluronan synthases (*HAS-1*, *HAS-2*, *HAS-3*) were quantified by qRT-PCR. Values are means ± SEM of two independent experiments, with significance referenced to both untreated and cytokine-treated controls (^##^ *p* < 0.01, ^###^ *p* < 0.001 vs. control; * *p* < 0.05, ** *p* < 0.01, *** *p* < 0.001 vs. TNF-α/IFN-γ-treated group). ECM, extracellular matrix; COL, collagen; HAS, hyaluronan synthase; qRT-PCR, quantitative real-time polymerase chain reaction.

To define the transcriptional basis of PB activity, the transcript levels of ECM-associated genes, including matrix metalloproteinases (MMPs), several collagen isoforms, and hyaluronan synthases, were quantified using qRT-PCR in HEKs exposed to TNF-α/IFN-γ. Combined cytokine stimulation strongly upregulated *MMP-1*, *MMP-2*, and *MMP-9* transcripts relative to unstimulated cells (^###^ *p* < 0.001; Figure 9), a pattern consistent with the engagement of matrix-degrading programs during the inflammatory response. These enzymes play critical roles in the breakdown of collagen and other structural components of the ECM, contributing to skin damage and loss of structural integrity.

In contrast, the expression levels of genes associated with ECM synthesis and maintenance were significantly reduced after cytokine stimulation. Specifically, collagen-related genes (*COL1A1*, *COL1A2*, *COL3A1*, and *COL4A1*), which are essential for maintaining dermal strength and elasticity, were markedly downregulated (^##^ or ^###^ *p* < 0.01–0.001 vs. control group). A parallel reduction was observed in the expression of hyaluronan synthase genes (*HAS-1*, *HAS-2*, and *HAS-3*), which drive hyaluronan biosynthesis and contribute to cutaneous moisture retention, indicating a broad impairment of ECM-building and water-holding capacity under inflammatory stress.

Notably, PB exposure dose-dependently blunted the TNF-α/IFN-γ-driven induction of MMPs (*** *p* < 0.001), consistent with its ability to limit matrix-degrading activity. Simultaneously, PB shifted the expression of collagen-related genes and hyaluronan synthases back toward baseline, indicating partial recovery of the cells’ ECM-synthetic capacity. Collectively, these results show that PB modulates both arms of ECM turnover, degradation and synthesis, at the transcriptional level.

Collectively, these data indicate that PB reshapes the transcriptional profile of ECM-related genes in TNF-α/IFN-γ-stimulated keratinocytes, supporting its role in preserving ECM homeostasis. Although these findings point to a plausible molecular basis for PB activity, the upstream signaling events driving these responses remain to be defined in future studies. ECM-related genes were analyzed at the transcriptional level to assess the coordinated regulation of MMPs, collagens, and hyaluronan synthases. The protein-level regulation of ECM responses was confirmed by ELISA-based quantification of MMP-1 and pro-collagen I α1 (Figure 3 and Figure 4), indicating that PB modulates ECM homeostasis at both transcriptional and protein levels.

## 3. Discussion

As the body’s first line of defense, the skin shields the underlying tissues from environmental insults such as UV radiation, pollutants, and mechanical stress [21]. The skin is organized into two principal compartments: the epidermis and dermis. The outermost epidermis is essential for retaining hydration and providing a protective interface against external stimuli, whereas the dermis, a thicker connective tissue layer rich in collagen, elastin, and vascular networks, confers mechanical strength, elasticity, and resilience to the skin [22]. The coordinated function of these two layers is critical for maintaining skin homeostasis and ensuring effective barrier function.

Skin aging stems from two distinct routes, one intrinsic and one extrinsic. Intrinsic aging is a time-dependent process governed by genetic and metabolic factors, marked by a gradual decline in fibroblast activity, collagen production, and skin hydration. In contrast, extrinsic aging is largely shaped by environmental stressors, of which UV radiation is the foremost [23]. Prolonged UV exposure drives excessive production of ROS, which perturbs the redox balance and imposes oxidative stress on skin cells. This oxidative stress activates multiple signaling pathways that promote inflammation and upregulate (MMPs, particularly MMP-1, which degrades type I and III collagen within the ECM [9,24,25]. Consequently, the structural integrity of the dermis is compromised, leading to wrinkle formation, loss of elasticity, and overall deterioration of skin architecture [26,27].

In addition to oxidative stress, inflammatory signaling is a major driver of accelerated skin aging. Pro-inflammatory cytokines, including TNF-α and IFN-γ, are known to worsen skin damage by boosting ROS production, driving MMP expression, and restraining ECM synthesis. The reciprocal reinforcement between oxidative stress and inflammation establishes a self-amplifying loop that further accelerates ECM degradation and impairs tissue repair. Compounds that can simultaneously act on oxidative stress, inflammation, and ECM balance are of particular interest for preventing and treating skin aging.

In this study, we systematically evaluated five polyphenolic compounds derived from *Nypa fruticans*—PA, HA, PB, CA, and EC—to identify the key bioactive component responsible for its protective effects. Across a series of comparative analyses, PB consistently showed the most pronounced activity in multiple functional assays, including ROS inhibition, reduced MMP-1 secretion, recovery of procollagen I α1 production, and attenuation of inflammatory responses.

PB markedly reduced intracellular ROS levels in TNF-α-stimulated HDFs, indicating its antioxidant capacity under inflammatory conditions. Although ROS levels were assessed using the DCFDA assay, which primarily detects general oxidative stress rather than specific ROSs, this method remains widely used for initial screening [28]. Nevertheless, future studies employing more advanced approaches, such as electron spin resonance (ESR) spectroscopy or organelle-specific ROS probes, would provide more detailed insights into the specific reactive species targeted by PB and the subcellular sites of its activity.

Consistent with its antioxidant effects, PB significantly suppressed TNF-α-induced MMP-1 secretion and restored pro-collagen I α1 production. These findings suggest that PB exerts a dual regulatory effect by inhibiting ECM degradation and promoting ECM synthesis. This coordinated regulation is particularly important for maintaining dermal integrity, as the balance between matrix degradation and synthesis determines the overall structural stability of the skin. In contrast, the other polyphenols tested in this study exhibited weaker or more selective effects, suggesting that PB may contribute substantially to the protective activity of *N. fruticans* under the present experimental conditions. This conclusion was further supported by radar plot analysis integrating multiple functional parameters, which identified PB as the compound exhibiting the most consistent overall activity among those evaluated.

Although PB has been previously reported to possess antioxidant and anti-inflammatory properties, the present study expands upon existing knowledge by directly comparing PB with other major polyphenolic constituents of *N. fruticans* under identical experimental conditions. Through this comparative approach, our findings provide evidence that PB may be a principal contributor to the protective effects of the extract and highlight its comparatively stronger activity across oxidative stress regulation, inflammatory responses, and ECM-related outcomes in human skin cell models.

Importantly, this study extends beyond fibroblast-based analyses by incorporating HEKs models, thereby providing a more comprehensive evaluation of the biological effects of PB across different skin cell types. Keratinocytes play a crucial role in initiating and propagating inflammatory responses in the skin, and their interaction with dermal fibroblasts is essential for maintaining the skin homeostasis. In TNF-α/IFN-γ-stimulated keratinocytes, PB significantly suppressed the secretion of pro-inflammatory cytokines, including IL-6, IL-8, and IL-1β. PB also reduced the production of inflammatory mediators, such as PGE_2_, COX-2, and NO. These findings indicate that PB effectively modulates inflammatory signaling pathways in epidermal cells, complementing its ECM-regulatory effects on dermal fibroblasts. These observations align with reports that natural compounds restrain inflammatory signaling in keratinocytes: an *Aster glehni* extract attenuated inflammatory responses while preserving skin-barrier molecules in human keratinocytes [29], fucoidan suppressed keratinocyte inflammation through inhibition of NF-κB and signal transducer and activator of transcription 1 (STAT) 1 signaling [30], and brazilin and taxifolin each lowered pro-inflammatory mediators such as IL-6 and IL-8 in TNF-α-stimulated keratinocytes [31,32].

At the transcriptional level, PB suppressed the TNF-α-induced upregulation of *MMP-1*, *MMP-2*, and *MMP-9*, while restoring the expression of key genes involved in ECM structure and hydration, including *COL1A1*, *COL1A2*, *COL3A1*, *COL4A1*, and hyaluronan synthase (*HAS-1*, *HAS-2*, and *HAS-3*). These gene expression changes suggest that PB contributes to the maintenance of ECM homeostasis not only by inhibiting degradation but also by enhancing the synthesis and functional properties of ECM components in the liver. In particular, the upregulation of hyaluronan synthases may be associated with improved skin hydration and barrier function, further supporting the potential of PB as a multifunctional skin-protective agent.

To clarify the molecular basis of these effects, the NF-κB and Nrf2 pathways were examined in TNF-α/IFN-γ-stimulated human keratinocytes. PB suppressed the phosphorylation of p65 and enhanced the nuclear accumulation of Nrf2 in a concentration-dependent manner, indicating that PB inhibits NF-κB activation while promoting Nrf2-mediated antioxidant signaling. Because NF-κB is a central transcriptional driver of pro-inflammatory cytokines, COX-2, and inducible nitric oxide production, and Nrf2 governs the cellular antioxidant defense, these findings provide a coherent mechanistic explanation for the concurrent reduction in inflammatory mediators and reactive oxygen species observed with PB. This dual regulation suggests that the protective activity of PB arises, at least in part, from the simultaneous restraint of inflammatory signaling and reinforcement of antioxidant response.

The superior bioactivity of PB compared to other polyphenols can be attributed, at least in part, to its chemical structure. PB is a dimeric proanthocyanidin composed of two flavan-3-ol units and carries numerous hydroxyl groups that can donate electrons and quench reactive oxygen species via resonance [33]. In contrast, simpler phenolic acids, such as PA and HA, possess fewer hydroxyl groups and lower redox capacities, which may explain their relatively modest antioxidant effects [34]. Furthermore, differences in molecular size, polarity, and stereochemistry may influence the cellular uptake, intracellular distribution, and metabolic stability of these compounds. For example, flavan-3-ols, such as CA and EC, are subject to stereoselective transport and enzymatic modification, which may affect their biological activity [35]. The dimeric structure of PB may confer enhanced stability and prolonged intracellular retention, contributing to its sustained biological effects.

Notwithstanding these encouraging results, several limitations warrant mention. First, the study relied on in vitro cell models, which cannot fully reproduce the complexity of the in vivo skin environment, where diverse cell types and systemic factors interact dynamically. Second, although the involvement of NF-κB and Nrf2 was confirmed by western blot analysis in keratinocytes, the contributions of other regulators, such as activator protein-1 (AP-1) and upstream mitogen-activated protein kinase (MAPK) signaling, remain to be elucidated. Third, the temporal dynamics of cellular responses were not characterized because time-course experiments were not performed. Future studies should resolve these gaps by incorporating in vivo models, pathway-specific assays, and time-resolved analyses to build a fuller picture of how PB acts. In addition, although protein-level evidence for ECM regulation was provided by ELISA quantification of secreted MMP-1 and pro-collagen I α1, protein-level validation was not extended to the entire panel of ECM-related genes. Broadening protein-level confirmation to additional ECM targets, such as MMP-9 and HAS2, represents a valuable direction for future studies. Direct quantification of mature type I collagen was also attempted using ELISA; however, reliable differences were not detected under the short-term two-dimensional culture conditions used here, likely reflecting the limited deposition of fibrillar collagen within this time frame. Approaches such as Sirius Red staining or macromolecular crowding–based assays may enable the more sensitive detection of mature collagen in future studies.

Overall, the present study identified procyanidin B1 as a key bioactive component of *Nypa fruticans,* capable of modulating oxidative stress, inflammatory responses, and ECM-related processes in human skin cells. By combining observations from both dermal fibroblasts and epidermal keratinocytes, this study provides a more integrated view of the protective effects of PB in the context of inflammation-associated skin aging. These findings support the use of PB as a functional ingredient in dermatological and cosmeceutical formulations aimed at preventing or mitigating skin aging. Additional research is needed to confirm these effects in vivo and to define the detailed molecular pathways underlying its activity.

## 4. Materials and Methods

### 4.1. Sample Preparation

**Figure 10:**
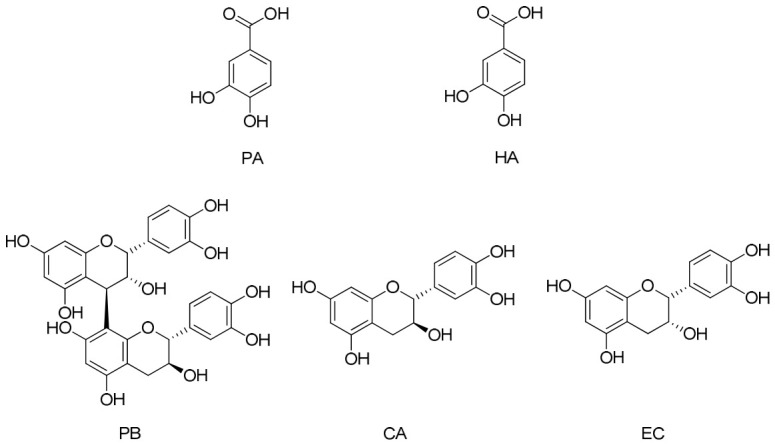
Chemical structures of the five major polyphenolic compounds identified in *N. fruticans* extract. PA, protocatechuic acid; HA, hydroxybenzoic acid; PB, procyanidin B1; CA, catechin; EC, epicatechin.

PA, HA, PB, CA, and EC used in this study were obtained from Sigma-Aldrich (Burlington, MA, USA), and their chemical structures are shown in Figure 10. Each compound was dissolved in 10 mM dimethyl sulfoxide (DMSO; Sigma-Aldrich, St. Louis, MO, USA) prior to use in the experiments, based on standard preparation protocols for polyphenolic compounds, as described in a previous study [19].

### 4.2. Cell Culture

Primary HDFs and HEKs sourced from PromoCell GmbH (Heidelberg, Germany) were propagated in Dulbecco’s Modified Eagle Medium (DMEM; Corning, Cat# 10-013-CV, Manassas, VA, USA), supplemented with 10% fetal bovine serum (FBS; Atlas, Cat# F-0500-A, Fort Collins, CO, USA) and 1% penicillin-streptomycin (Gibco, Cat# 15140122, Grand Island, NY, USA). Cells were incubated at 37 °C in a 5% CO_2_ humidified atmosphere to ensure proper proliferation and physiological conditions, following the established protocols for culturing primary fibroblasts [36].

### 4.3. Cell Viability

To assess the cytotoxicity of the test compounds, HDFs were seeded in 96-well plates (SPL Life Sciences, Cat# 30096, Pocheon, Republic of Korea) at a density of 1 × 10^4^ cells per well and incubated at 37 °C for 24 h. Following this initial incubation, the culture medium was exchanged for serum-free DMEM to induce nutrient deprivation, and the cells were maintained under the same conditions for an additional 24 h. After incubation, 100 μL of 10% EZ-Cytox solution (DoGenBio, Cat# EZ-1000, Seoul, Republic of Korea) was added to each well. Absorbance at 450 nm was recorded after 1 h using a microplate reader (SPARK 10M, Tecan, Männedorf, Switzerland) to quantify cell viability, following established colorimetric assay protocols for cytotoxicity assessment [37].

### 4.4. ROS Assay

To assess intracellular ROS accumulation, HDFs were plated in black-walled 96-well microplates (Corning, Cat# 3603, Corning, NY, USA) at a density of 1 × 10^4^ cells per well and incubated for 24 h at 37 °C in a humidified incubator. The culture medium was then replaced with serum-free DMEM to induce cellular stress, and the cells were incubated for an additional 24 h. Subsequently, the cells were exposed to the test compounds at non-toxic concentrations for 1 h. Subsequently, 20 ng/mL TNF-α (PeproTech, Cat# 300-01A, Rocky Hill, NJ, USA) and 10 μM of the ROS-sensitive probe 2′,7′-dichlorofluorescein diacetate (DCFDA; Sigma-Aldrich, Cat# D6883, St. Louis, MO, USA) was added, and the cells were incubated for 15 min. Post-treatment, the cells were gently rinsed with Dulbecco’s phosphate-buffered saline (DPBS; Welgene, Cat# LB 004-02, Gyeongsan, Republic of Korea), and the fluorescence signal was detected using a microplate reader (SPARK 10M, Tecan, Männedorf, Switzerland) at 485 nm excitation and 535 nm emission wavelengths, following previously established protocols for quantifying ROS in fibroblasts [38].

### 4.5. Enzyme-Linked Immunosorbent Assay (ELISA)

HDFs and HEKs were seeded into 48-well culture plates (SPL Life Sciences, Cat# 30048, Pocheon, Republic of Korea) at a density of 2 × 10^4^ cells/well and incubated for 24 h under standard conditions to allow proper cell adherence. The medium was then replaced with serum-free DMEM to initiate nutrient deprivation, and the cells were cultured for 24 h. After the starvation phase, the cells were treated with various concentrations of the test compounds deemed non-cytotoxic, followed by incubation for 1 h. TNF-α (20 ng/mL) was subsequently added, and the cells were incubated for 12 or 24 h, as specified in the experimental timeline. Following treatment, cell culture supernatants were collected and analyzed using a commercially available sandwich ELISA. Secreted levels were quantified using commercially available sandwich ELISA kits (R&D Systems, Minneapolis, MN, USA): MMP-1 (Cat# DY901B), pro-collagen I α1 (Cat# DY6220-05), IL-6 (Cat# DY206), IL-8 (Cat# DY208), IL-1β (Cat# DY201), PGE_2_ (Cat# KGE004B), and COX-2 (Cat# DYC4198-2), according to the manufacturers’ instructions. Absorbance readings were obtained at 450 nm using a microplate reader (SPARK 10M; Tecan, Männedorf, Switzerland), as previously described [39].

### 4.6. Integrated Analysis and Radar Plot Visualization

To compare the overall efficacy of the five major polyphenols, an integrated analysis was performed using radar plot visualization based on three biological parameters: inhibition of intracellular ROS production, suppression of MMP-1 secretion, and restoration of pro-collagen I α1 secretion. Radar plots were generated using Microsoft Excel (version Microsoft 365; Microsoft Corporation, Redmond, WA, USA).

For each parameter, the observed values of all tested compounds were arranged according to their relative efficacy and normalized by dividing the value range between the minimum and maximum responses into four equal intervals. Each compound was assigned an ordinal score ranging from 1 to 4 according to the interval into which its value fell, with higher scores indicating stronger biological activity. The final integrated score for each compound was calculated as the mean of the three parameter scores and used to generate a radar plot. Larger polygonal areas represented greater overall efficacy across the evaluated parameters.

### 4.7. Nitric Oxide (NO) Assay

HEKs were cultured in 96-well plates at a seeding density of 1 × 10^4^ cells/well and incubated at 37 °C with 5% CO_2_ for 24 h. The medium was then switched to a serum-free formulation to simulate nutrient-deficient conditions, and the cells were incubated for an additional 24 h. Subsequently, the cells were treated with test samples at concentrations confirmed to be non-cytotoxic for 1 h before stimulation with 20 ng/mL TNF-α for 24 h. After the treatment period, NO production was estimated indirectly by measuring nitrite accumulation in the culture supernatant using the Griess reagent (Promega, Cat# G2930, Madison, WI, USA). Equal volumes of the collected supernatant and freshly prepared Griess reagent mixture, comprising 1% sulfanilamide, 0.1% *N*-(1-naphthyl)ethylenediamine, and 5% phosphoric acid, were combined and incubated at ambient temperature for 10 min. The resulting colorimetric change was evaluated by recording the absorbance at 540 nm using a microplate reader (SPARK 10M; Tecan, Männedorf, Switzerland), as described previously [40].

### 4.8. Western Blot Analysis

HEKs were plated in 6-well plates (SPL Life Sciences, Cat# 30006, Pocheon, Republic of Korea) at 3 × 10^5^ cells/well and allowed to attach for 24 h, after which the medium was replaced with serum-free DMEM overnight. The cells were pretreated with PB (0.3, 1, or 3 μM) for 1 h prior to stimulation with TNF-α (20 ng/mL) and IFN-γ (20 ng/mL; PeproTech, Cat# 300-02, Rocky Hill, NJ, USA). For NF-κB signaling analysis, whole-cell lysates were prepared in RIPA buffer (Thermo Fisher Scientific, Cat# 89900, Waltham, MA, USA) 30 min after stimulation. For Nrf2 analysis, nuclear fractions were isolated using a nuclear/cytoplasmic extraction kit (Thermo Fisher Scientific, Waltham, MA, USA) 2 h after stimulation. Lysates were clarified by centrifugation (13,000 rpm, 10 min, 4 °C), and protein concentrations were determined using a Pierce™ BCA Protein Assay Kit (Thermo Fisher Scientific, Waltham, MA, USA). Equal amounts of protein were resolved by SDS–PAGE and transferred onto PVDF membranes (Millipore, Bedford, MA, USA). After blocking with 5% skim milk in TBS-T, the membranes were probed overnight at 4 °C with the following primary antibodies from Cell Signaling Technology (Danvers, MA, USA): Phospho-NF-κB p65 (Ser536) (93H1) Rabbit mAb (Cat# 3033), NF-κB p65 (D14E12) XP Rabbit mAb (Cat# 8242), NRF2 (D1Z9C) XP Rabbit mAb (Cat# 12721), Lamin B1 (D9V6H) Rabbit mAb (Cat# 12586), and GAPDH (D16H11) XP Rabbit mAb (Cat# 5174). The membranes were subsequently incubated with an HRP-linked anti-rabbit IgG secondary antibody (Cell Signaling Technology, Cat# 7074) for 2 h at room temperature. Immunoreactive bands were visualized using SuperSignal™ West Femto substrate (Thermo Fisher Scientific, Waltham, MA, USA) and quantified using ImageJ (version 1.53k; National Institutes of Health, Bethesda, MD, USA). Phospho-p65 was normalized to total p65, and nuclear NRF2 levels were normalized to Lamin B1 levels. GAPDH was used as the loading control for whole-cell lysates.

### 4.9. Quantitative Real-Time Polymerase Chain Reaction (qRT-PCR)

HEKs were seeded at a density of 3 × 10^5^ cells per well in 6-well culture plates and incubated for 24 h to ensure sufficient adhesion. To induce nutrient stress, the growth medium was replaced with DMEM lacking fetal bovine serum, and the cells were maintained for overnight. On the following day, the cells were exposed to the designated test compounds for 1 h, followed by stimulation with 20 ng/mL TNF-α for 12 h, depending on the target gene being analyzed.

Total RNA was isolated using the RNeasy Mini Kit (Qiagen, Cat# 74104, Germantown, MD, USA), and complementary DNA (cDNA) was synthesized using the RevertAid First Strand cDNA Synthesis Kit (Thermo Fisher Scientific, Cat# K1622, Waltham, MA, USA), according to the manufacturer’s instructions. qRT-PCR was performed using the PowerUp™ SYBR™ Green Master Mix (Applied Biosystems, Cat# A25742, Austin, TX, USA) on a QuantStudio 3 Real-Time PCR system (QuantStudio 3, Applied Biosystems, Foster City, CA, USA). The specific primer sequences used are listed in Table 1. Primers were synthesized by Bioneer (Daejeon, Republic of Korea). Thermal cycling consisted of an initial incubation at 50 °C for 2 min and pre-denaturation at 95 °C for 10 min, followed by 40 cycles of 15 s at 95 °C and 1 min at 60 °C. Melt curve analysis was performed at the end of the run, consisting of steps at 95 °C for 15 s, 60 °C for 1 min, and 95 °C for 15 s, as described previously [19]. Relative mRNA expression levels were normalized to *β-actin* as the endogenous control and calculated using the comparative 2^−ΔΔCT^ method.

### 4.10. Statistical Analyses

Statistical evaluations were conducted using GraphPad Prism (v8.0.1; GraphPad Software Inc., La Jolla, CA, USA). Differences among experimental groups were analyzed using one-way ANOVA, and Tukey’s multiple comparison test was used for post hoc analysis. A threshold of *p* < 0.05 was used to define statistical significance across all comparisons, as described previously [40].

## 5. Conclusions

Taken together, the present study establishes PB, the principal polyphenol of *Nypa fruticans*, as a protective agent against inflammation-linked oxidative and ECM damage in human skin cells. In dermal fibroblasts challenged with TNF-α, PB reduced the accumulation of intracellular ROS, curbed MMP-1 secretion, and promoted procollagen I α1 production, indicating a regulatory role in both ECM breakdown and synthesis.

PB also tempered inflammatory responses in keratinocytes co-stimulated with TNF-α and IFN-γ, reducing the release of pro-inflammatory cytokines (IL-6, IL-8, and IL-1β) and mediators such as PGE_2_, COX-2, and NO. At the transcriptional level, PB downregulated ECM-degrading enzymes (*MMP-1*, *MMP-2*, and *MMP-9*) and upregulated collagen- and hyaluronan-associated genes (*COL1A1*, *COL1A2*, *COL3A1*, *COL4A1*, *HAS1*, *HAS2*, and *HAS3*), supporting its role in maintaining ECM homeostasis and skin hydration.

Collectively, these findings indicate that PB modulates oxidative stress, inflammatory responses, and ECM-related processes in human skin cells, at least in part, through the inhibition of NF-κB and activation of Nrf2 signaling, underscoring its candidacy as a functional cosmeceutical component for mitigating inflammation-associated skin aging. However, further studies incorporating in vivo models and mechanistic analyses are required to validate its biological relevance and elucidate its underlying molecular pathways.

## Figures and Tables

**Table 1:** Primer sequences.

Gene	Sense Primer Sequence (5′-3′)	Antisense Primer Sequence (5′-3′)
*MMP-1*	ATTCTACTGATATCGGGGCTTT	ATGTCCTTGGGGTATCCGTGTA
*MMP-2*	CAGGGAATGAGTACTGGGTCTATT	ACTCCAGTTAAAGGCAGCATCTAC
*MMP-9*	CACTGTCCACCCCTCAGAGC	CACTTGTCGGCGATAAGG
*COL1A1*	CTCGAGGTGGACACCACCCT	CAGCTGGATGGCCACATCGG
*COL1A2*	AGAAACACGTCTGGCTAGGAG	GCATGAAGGCAAGTTGGGTAG
*COL3A1*	GTTTTGCCCCGTATTATGGA	GGAAGTTCAGGATTGCCGTA
*COL4A1*	ACTCTTTTGTGATGCACACCA	AAGCTGTAAGCGTTTGCGTA
*HAS-1*	CCACCCAGTACAGCGTCAAC	CATGGTGCTTCTGTCGCTC
*HAS-2*	TTTGTTCAAGTCCCAGCAGC	ATCCTCCTGGGTGGTGTGAT
*HAS-3*	CCCAGCCAGATTTGTTGATG	AGTGGTCACGGGTTTCTTCC
*β-actin*	AGAGATGGCCACGGCTGCTT	ATTTGCGGTGGACGATGGAG

## Data Availability

The data presented in this study are available within the article.

## Abbreviations

ANOVA	Analysis of variance
AP-1	Activator protein-1
BCA	Bicinchoninic acid
CA	Catechin
cDNA	Complementary DNA
COL1A1	Collagen type I alpha 1
COL1A2	Collagen type I alpha 2
COL3A1	Collagen type III alpha 1
COL4A1	Collagen type IV alpha 1
COX-2	Cyclooxygenase-2
DCFDA	2′,7′-Dichlorofluorescein diacetate
DMEM	Dulbecco’s Modified Eagle Medium
DMSO	Dimethyl sulfoxide
DPBS	Dulbecco’s phosphate-buffered saline
EC	Epicatechin
ECM	Extracellular matrix
ELISA	Enzyme-linked immunosorbent assay
FBS	Fetal bovine serum
GAPDH	Glyceraldehyde-3-phosphate dehydrogenase
HA	Hydroxybenzoic acid
HAS	Hyaluronan synthase
HDFs	Human dermal fibroblasts
HEKs	Human epidermal keratinocytes
HRP	Horseradish peroxidase
IFN-γ	Interferon-γ
IL	Interleukin
mAb	Monoclonal antibody
MAPK	Mitogen-activated protein kinase
MMP	Matrix metalloproteinase
NF-κB	Nuclear factor kappa B
NO	Nitric oxide
Nrf2	Nuclear factor erythroid 2–related factor 2
PA	Protocatechuic acid
PB	Procyanidin B1
PGE_2_	Prostaglandin E2
p-p65	Phosphorylated p65
PVDF	Polyvinylidene fluoride
QC	Quercetin
qRT-PCR	Quantitative real-time polymerase chain reaction
RIPA	Radioimmunoprecipitation assay
ROS	Reactive oxygen species
SDS-PAGE	Sodium dodecyl sulfate–polyacrylamide gel electrophoresis
SEM	Standard error of the mean
SOD	Superoxide dismutase
STAT1	Signal transducer and activator of transcription 1
TNF-α	Tumor necrosis factor-α
UV	Ultraviolet
